# Economic Aspects of Infertility Care in Iran

**DOI:** 10.34172/aim.2022.112

**Published:** 2022-10-01

**Authors:** Abdoreza Mousavi

**Affiliations:** ^1^Department of Health Economics and Management, School of Public Health, Tehran University of Medical Sciences, Tehran, Iran

## Dear Editor,

 The realization of the desire for fertility is a fundamental human right linked to the International Conference on Population and Development (ICPD) call to action and sustainable development goals (SDGs).^1^ Infertility is among the most significant global and ongoing health issues, affecting millions of people around the world.^2,3^ It is estimated that between 8% and 12% of reproductive-age couples worldwide are affected by infertility.^3^ In Iran, the lifetime prevalence of primary and secondary infertility based on clinical definition is %11.9 and %15.3 respectively.^4^

 Over the last few decades, infertility care has improved and several costly treatments, such as assisted reproductive techniques (ART), have become available for clinical use.^5^ However, there are still major problems with access, availability, quality, and cost of infertility interventions.^1,2^ According to expert opinion, infertility treatment is a complex, expensive, and time-consuming process. In addition to the cost of diagnostic tests and the main intervention, medicine and medical consumables make up a significant portion of the expenses, and its cost depends on exchange rate fluctuations.

 Among the duties of health system is providing quality health care and financial protection for population against catastrophic health expenditure and impoverishment. Infertility treatment, may lead to catastrophic health expenditure and economic instability.^6,7^ For example, a study in South Africa showed that 22% of infertile couples who underwent ARTs, incurred catastrophic expenditures. Nevertheless, some families tend to pay the high cost of infertility treatment to have a child, and even poor couples are willing to endure financial hardship.^7^ This is a demonstration of how important fertility is for households and the society.

 Government policies can reduce many inequities in access to reproductive care^1^; as comprehensive insurance coverage can reduce the disparities in access to infertility care. Without broad insurance coverage for assisted reproductive techniques, infertility is implicitly recognized as a disease not deserving financial support, and preventing many patients from accessing infertility care.^8^

 Addressing the issue of infertility is a priority in Iran, where the prevalence of primary infertility is higher than the global average, and the population is aging with increasing speed; the population over the age of 60 and 65 in Iran will increase to 31% and 22% in 2050, respectively.^9^ Though a recent shift in population policies towards increasing the birth rate have prompted infertility treatment policies to make their way into the health sector agenda,^10^ the economic aspects of infertility have received less attention.

 Infertility threatens the fundamental right to procreation. It has psychological, social and economic consequences for infertile couples, and in the long run, affects the population, workforce and macroeconomic factors by changing the demographic structure of a society. Therefore diagnosis and treatment of infertility should be prioritized in national population and development policies.

 Given the growing trend of population aging and the realization of the “*family protection and youth policy*” goals, it seems that financial support for infertile couples who want to have children is an effective option. The following measures are therefore recommended:

- Establishing an integrated system to collect up-to-date, valid, and high-quality data on the cost and frequency of use of different infertility treatments, as well as demographic data of infertile couples.
- Continuous monitoring of collected data to sustain awareness around the costs of infertility treatment allowing the allocation of appropriate financial resources.
- Continuity in providing, enhancement and expansion of insurance coverage including medical services, medical consumables and medications.
- Achieving sustainable finance to address the growth of expenses as well as the increase of infertility couples applying for support services.
- Monitoring service quality and coverage to increase the continuity of adherence to therapy and the effectiveness of treatment.
